# NIATx-TI versus typical product training on e-health technology implementation: a clustered randomized controlled trial study protocol

**DOI:** 10.1186/s13012-020-01053-4

**Published:** 2020-10-23

**Authors:** Veronica M. White, Todd Molfenter, David H. Gustafson, Julie Horst, Rachelle Greller, David H. Gustafson, Jee-Seon Kim, Eric Preuss, Olivia Cody, Praan Pisitthakarm, Alexander Toy

**Affiliations:** 1 Department of Industrial and Systems Engineering, University of Wisconsin-Madison, 1513 University Ave, Madison, WI 53706 USA; 2 Department of Educational Psychology, University of Wisconsin-Madison, Educational Sciences, 1025 West Johnson St, Madison, WI 53706-1706 USA; 3 Division of Behavioral Health, Iowa Department of Public Health, Lucas State Office Building, 321 E. 12th Street, Des Moines, IA 50319-0075 USA

**Keywords:** Evidence-based practice implementation, Mobile technology, Technology implementation model, Coaching, Substance use disorder treatment

## Abstract

**Background:**

Substance use disorders (SUDs) lead to tens-of-thousands of overdose deaths and other forms of preventable deaths in the USA each year. This results in over $500 billion per year in societal and economic costs as well as a considerable amount of grief for loved ones of affected individuals. Despite these health and societal consequences, only a small percentage of people seek treatment for SUDs, and the majority of those that seek help fail to achieve long-term sobriety. E-health applications in healthcare have proven to be effective at sustaining treatment and reaching patients traditional treatment pathways would have missed. However, e-health adoption and sustainment rates in healthcare are poor, especially in the SUD treatment sector. Implementation engineering can address this gap in the e-health field by augmenting existing implementation models, which explain organizational and individual e-health behaviors retrospectively, with prospective resources that can guide implementation.

**Methods:**

This cluster randomized control trial is designed to test two implementation strategies at adopting an evidence-based mobile e-health technology for SUD treatment. The proposed e-health implementation model is the Network for the Improvement of Addiction Treatment–Technology Implementation (NIATx-TI) Framework*.* This project, based in Iowa, will compare a control condition (using a typical software product training approach that includes in-person staff training followed by access to on-line support) to software implementation utilizing NIATx-TI, which includes change management training, followed by coaching on how to implement and use the mobile application. While e-health spans many modalities and health disciplines, this project will focus on implementing the Addiction Comprehensive Health Enhancement Support System (A-CHESS), an evidence-based SUD treatment recovery app framework. This trial will be conducted in Iowa at 46 organizational sites within 12 SUD treatment agencies. The control arm consists of 23 individual treatment sites based at five organizations, and the intervention arm consists of 23 individual SUD treatment sites based at seven organizations

**Discussion:**

This study addresses an issue of substantial public health significance: enhancing the uptake of the growing inventory of patient-centered evidence-based addiction treatment e-health technologies.

**Trial registration:**

ClinicalTrials.gov, NCT03954184. Posted 17 May 2019

Contributions to the literature

- Research suggests that e-health technologies in substance use treatment are promising, yet only recently have agencies started to use them.
- This study suggests a technology implementation model for incorporating e-health technologies in substance use disorder treatment agencies.
- This randomized control trial addresses a gap in the current literature by comparing the effectiveness of an evidence-based implementation strategy (NIATx-TI) with a widely used implementation strategy (Product Training). It will also be the first to do so in implementing A-CHESS, an evidence-based e-health framework.
- The outcomes of this study are extremely relevant given the uptick in interest for e-health technologies post COVID-19.

## Background

Every day on average 96 Americans die due to opioid overdoses, and another six die due to alcohol poisoning, making substance use disorders (SUDs) the third-leading cause of preventable death in the USA [1]. Many injuries and diseases (e.g., cancer, diabetes, cardiovascular problems, cirrhosis, and HIV/AIDS) are caused or exacerbated by substance misuse [2]. Substance misuse results in tens-of-thousands of overdose deaths and other forms of preventable deaths [3] and more than $500 billion in societal and economic costs each year [4]. Despite the health and societal consequences of SUDs, just 9.2% of those who need treatment receive it [5], and 75% of those treated fail to achieve long-term sobriety [6]. New, more efficient approaches are required to fill the gaps in both the use and effectiveness of SUD treatment.

Patient-centered mobile e-health technologies offer innovative ways to improve treatment and recovery supports for SUDs [7]. In February 2018, 81% of the adult population and 92% of the persons aged 18–34 in the USA owned a smartphone [8]. Additionally, 75% of US adults below the median US income level owned a smartphone [8]. Several meta-analyses of mobile behavioral e-health interventions have found superior treatment outcomes when compared to a control group [9–14]. The combination of consumer interest and research evidence will continue to place positive pressure on the use of e-health. In this study, we use a mobile application because daily mobile technology use is nearly ubiquitous.

Despite the promise of e-health technologies, the benefits are far from being realized [15–17]. A prominent example of this is in SUD treatment services. Despite the availability of evidence-based e-health tools such as the Drinker’s Check-up [18, 19], Therapeutic Education System (TES) [20], Computer-Based Training for Cognitive Behavioral Therapy (CBT4CBT) [21], and Addiction Comprehensive Health Enhancement Support System (A-CHESS) [22], a 2012 survey showed all of these technologies together were used by < 1% of SUD treatment providers [23]. Since the onset of COVID-19, researchers and practitioners have focused on e-health technologies to deliver virtual SUD services while in-person treatment has become limited [24–26]. This study tests whether an evidence-based technology implementation framework can reduce the gap between patient-centered e-health evidence and practice.

Extensive research supports building models to speed diffusion and sustain technology adoption [27]. Technology adoption research began with models of behavioral change [28] that explained why users abandon traditional practices in favor of new technologies [29]. Over time, the use of these models has expanded due to the fundamental role organizational practices and climate play into individual decisions to adopt and continue to use a technology [30, 31]. These newer frameworks include the key organizational factors of management support, clinician satisfaction, clinical workflow, and financial resources for technology purchase, implementation, and use [32, 33]. Financial resources such as start-up grants and reimbursement supporting technology use are important factors in technology adoption. However, reviews by Brooks et al. [32] and Jeyaraj et al. [33] demonstrate that reimbursement cannot be the only issue considered. Many models describe the barriers in technology adoption [34, 35], but not how to address them. This study examines the competencies and processes that remained unexplored in previous technology implementation research to help health centers and patients participate actively in mobile e-health.

The organizational planning field has used two relatively stable theoretical planning approaches for the past two decades: deliberate/prescriptive planning and emergent/descriptive planning [36]. Porter’s Five Forces Framework [37] created a foundation for deliberate planning. His “look before you leap” planning approach relies on a centralized planning function and development of “the plan”. Concerns with this methodology led Mintzberg and Waters [38] to assert that Porter’s *deliberate* (sometimes called *prescriptive*) *planning* for organizational strategy and change was necessary, but not sufficient. Simply too much occurred after planning to create a single “plan”. They instead developed an *emergent* (sometimes called *descriptive*) *planning* approach, which used data that emerged from the implementation process to improve the plan throughout implementation. This approach moved planning from a purely centralized (top-down) process to include a more emergent (staff engaged) approach [39–41]. These concepts from the literature of pre-implementation (*deliberate*) and post-implementation (*emergent*), along with our authors’ field experiences [42–45], lead to the creation of the Network for the Improvement of Addiction Treatment–Technology Implementation (NIATx-TI) framework.

NIATx-TI was piloted in the Iowa Rural Health Information Technology Initiative (IRHIT) with 14 of Iowa’s 105 SUD treatment sites and resulted in a twofold increase in patients receiving virtual treatment. The framework’s *deliberate* component includes using an organizational change management assessment (OCM) [46]. This tool identifies assets and barriers to technology implementation and adoption. Results from the OCM can be incorporated into the technology’s implementation protocol. The framework’s *emergent* component includes using a project team to uncover and prioritize implementation barriers as they arise, develop changes to address identified barriers, and monitor selected adoption measures while receiving monthly coaching.

This research will test the technology adoption framework (NIATX-TI) in increasing the use of the Addiction Comprehensive Health Enhancement Support System (A-CHESS), an evidence-based technology platform developed for treating SUD [22, 47–51]. A-CHESS is based on the CHESS platform, which has been studied in 16 randomized control trials (RCTs) across several technology platforms [52–54]. These RCTs, each involving hundreds of people, found that the CHESS platform improved key dimensions of quality of life [52, 55], health behavior adherence (e.g., smoking cessation and risky drinking) [56–58], and outcomes (e.g., lung cancer death rates and sobriety). A-CHESS is consistent with well-respected health behavioral change models such as self-determination theory (SDT) [59] and Marlatt’s Cognitive Behavior model [60] of relapse prevention. The CHESS platform has also been an effective method for clinicians to interact with their patients [61]. For example, A-CHESS provides a clinician dashboard that provides information about worrisome changes in symptoms collected from the patient during self-monitoring of risk-related items (i.e., sleeping problems, depression, urge, risky situation, and relationship troubles) [62]. The dashboard is intended to bring clinician attention to emerging symptoms quickly. A-CHESS keeps patients in treatment longer by bridging the gap between face-to-face SUD treatment sessions, through clinic communications, peer-support, and having reliable and vetted resources accessible 24/7 [22, 48–50]. The Recovering Iowans Supporting Each other (RISE-Iowa) mobile application is an A-CHESS technology, as it contains all of the features of an A-CHESS platform. Having a mobile application dedicated to this study will allow for a controlled on-line discussion environment consisting of only consenting patients from participating organizations.

Product training and on-line support have been the customary approach to technology adoption [63, 64]. In this two-arm study, we will compare a control condition (using a typical product training approach to software implementation followed by access to on-line support) to the intervention condition (the typical product training combined with NIATx-TI). Iowa was selected as the test site for this proposed study. Cluster randomization was used in order to have similar organizations, in terms of the assessed criteria, in both study arms.

## Methods

### Study objectives

The primary aim of this cluster randomized controlled trial (RCT) is to compare the effectiveness of NIATx-TI (arm 2 only) and product training in implementing an evidence-based platform (A-CHESS), via RISE-Iowa. Impact will be measured using the RE-AIM framework [65]: *Reach or Participation (Primary Outcome)*: (a) percent and representativeness, based on eligible patients who create an account on the RISE-Iowa app, and (b) average RISE-Iowa use, by days of use; *Effectiveness*: treatment retention rates of sites between the two arms; *Adoption*: percent and representativeness, based on age, gender, and education level of counselors using RISE-Iowa; *Implementation*: (a) organizational readiness for, and (b) fidelity of RISE-Iowa implementation and (c) qualitative analyses to develop a better understanding of the implementation processes for delivering NIATx-TI compared to product training/on-line support alone; *Maintenance*: RISE-Iowa use (e.g., number of RISE-Iowa accounts and days of use) during the sustainability phase. All objectives will be compared at the cluster level.

### Trial design

This research extends over 30 months (18M test period and12M sustainability period). We are using a mixed-methods approach to compare the effectiveness of the *control*: product training alone (arm 1) and *intervention*: product training + NIATx-TI (arm 2) to increase RISE-Iowa use among adults (> 18 years) receiving outpatient SUD care. Both the intervention and control arms will complete all the same data collection instruments used in this study. A cluster is a pair of organizations that have similar criteria and will be randomly placed in opposite arms of the study.

### Participants

We selected Iowa as a partner because of its mix of urban and rural settings, the presence of minority populations, the strength of its data collection and reporting systems, and the limited use of A-CHESS platforms.

**Fig. 1:**
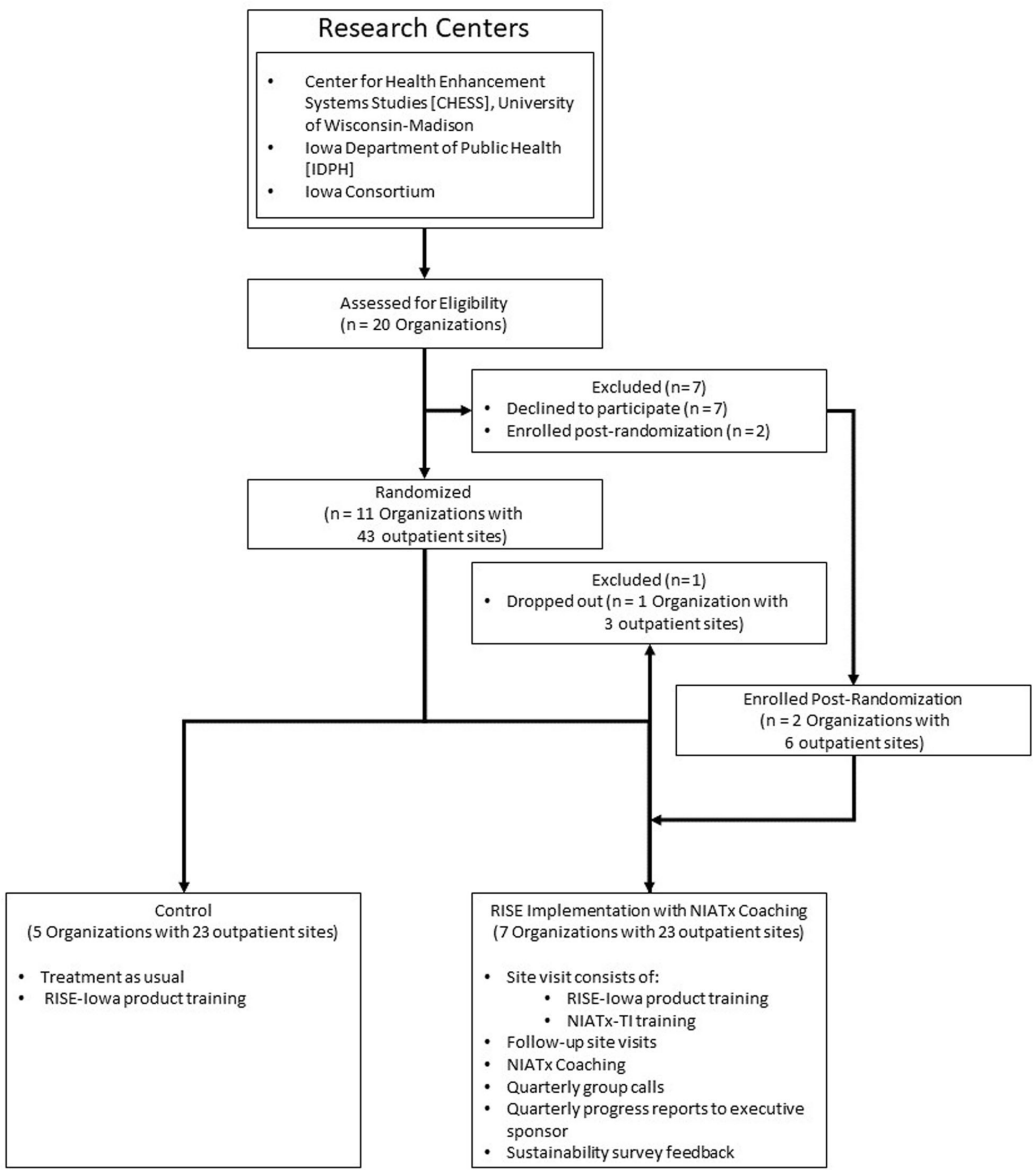
Consort diagram: RISE-Iowa study recruitment

Recruitment focused on organizations funded by the Iowa Department of Public Health (IDPH) and licensed to provide outpatient treatment services. We assessed eligible organizations for their readiness for implementation, number of outpatient sites, and mix of urban and rural populations. Each Iowa SUD organization consists of one to seven outpatient sites. The aim was to enroll 40 addiction treatment sites with 20 in each arm and estimated this would require 16 organizations. Of the 20 eligible organizations, 11 Iowa organizations consisting of 43 addiction treatment centers were initially enrolled in this study. Using cluster randomization, we randomized the 11 organizations into two groups: five organizations with 23 addiction treatment centers into the control arm and six organizations with 20 addiction treatment centers into the intervention arm. Post-randomization, one organization in the intervention arm dropped out of the study, and two organizations enrolled post-randomization. The two new organizations were assigned to the intervention arm to achieve a roughly equal number of control and experimental sites. Figure 1 shows the CONSORT diagram, which has a total of 12 organizations with 46 addiction treatment centers, with five organizations with 23 addiction treatment centers in the control arm and seven organizations with 23 addiction treatment centers in the intervention arm.

### Randomization

The first step of the stratified randomization (or blocking with matched pairs) was to determine sample matchings for the 11 organizations that originally agreed to participate. The matching of the organizations depended on (a) readiness for technology adoption score (assessed by IDPH based on a previous web portal implementation study), (b) the number of rural sites, and (c) the number of outpatient sites per organization. We applied urn randomization to achieve arm balance, using these three traits. Additionally, the odd number of organizations in the study (*n* = 11) created one matched pair with two intervention organizations and one control organization. Blinding or concealment of the participants and researchers was not logistically feasible. Post-randomization, a second matched pair was created that paired two intervention organizations to one control organization, to account for the late enrollment of one of the organizations. The second late enrollment replaced the organization that had dropped out of the intervention that was a part of the first 2:1 matched pair. These non-randomized additions were made to keep the total number of SUD treatment centers in each arm even and the pairs as equal as possible in terms of the original randomization matching criteria.

### Staff and patient recruitment and consent procedures

A member of the management team at each participating organization completed Klein’s Financial Resource Availability Inventory [66] and the Organizational Change Manager (OCM) for leadership survey [46]. Additionally, a site leader at each location identified up to seven staff to complete the OCM. No staff member at the organization had access to the survey data, nor have they received any data from the surveys. The consent form that precedes the surveys states that completing the survey infers their consent. For those who do not complete the survey, the survey will not be recorded, and no documentation of those who decline to participate. Staff completing surveys are treated as human subjects, informed of possible risks, offered the opportunity to decline participation in the survey, and afforded the protections of our data safety and monitoring plan.

All outpatient counselors and peer recovery support specialists at participating organizations were invited to be part of the study. All clients at our participating organizations with a SUD, > 18 years of age, who understand English, and have access to a smartphone, are introduced to RISE-Iowa during regular treatment at the participating organization. In both arms of the study, counselors give the patient their site location’s unique code to create an account on the RISE-Iowa mobile application. Each organization will create their own protocol for introducing patients to RISE-Iowa. Therefore, how counselors and staff introduce RISE-Iowa to patients and disseminate their site’s unique code for patients to create an account on the app will differ between organizations and potentially between sites within an organization. Once patients are introduced to RISE-Iowa, they can download the mobile application through the Apple Store or Google Play Store. For this study, patient consent is part of the account creation process when the patient downloads and registers on RISE-Iowa. Patients are enrolled in the study after they download RISE-Iowa, create an account, and verify their informed consent.

### Interventions

The *control arm* for this study includes:

1. *RISE-Iowa demo for executive leadership* with clinical leadership and conducted by the RISE-Iowa product trainer takes place during the project setup phase. This initial RISE-Iowa demo (a) describes the features of RISE-Iowa (tutorial), (b) explains the study design and stipend information, (c) informs leadership how to choose an appropriate RISE-Iowa site coordinator for each site location, and (d) brings understanding to our team about how the organization operates and if special considerations are needed.
2. *RISE-Iowa demo for site leaders* with the organization’s IT, RISE-Iowa site coordinator, and conducted by the RISE-Iowa product trainer takes place during the project setup phase, after the initial RISE-Iowa demo. This secondary RISE-Iowa demo (a) describes the features of RISE-Iowa (tutorial), (b) provides RISE-Iowa organizational case examples of how it is implemented, and (c) provides examples of how to recruit and prepare clinicians and patients for RISE-Iowa.
3. *RISE-Iowa product training* is conducted by the RISE-Iowa product trainer and an IT assistant. The product training occurs at the organizational sites. The training serves as the start date of that organizations’ 18-month intervention period. All interested counselors and staff at the organization are invited to participate in the product training, which consists of (a) meeting with organizational leadership to discuss RISE-Iowa implementation plan after the on-site visit, (b) demonstrating RISE-Iowa features and how RISE-Iowa allows patients to self-manage their recovery in conjunction with their counselors, (c) demonstrating how to use the RISE-Iowa clinician dashboard, and (d) providing promotional brochures, posters, flyers.
4. *On-line technical support* will occur for the 18 months following the product training. Patients and counselors can call or e-mail questions regarding the RISE-Iowa mobile application. Some examples include password/account recovery, account deletion, bug reporting, notification of inappropriate app use, and suggestions for improving the app.

**Fig. 2:**
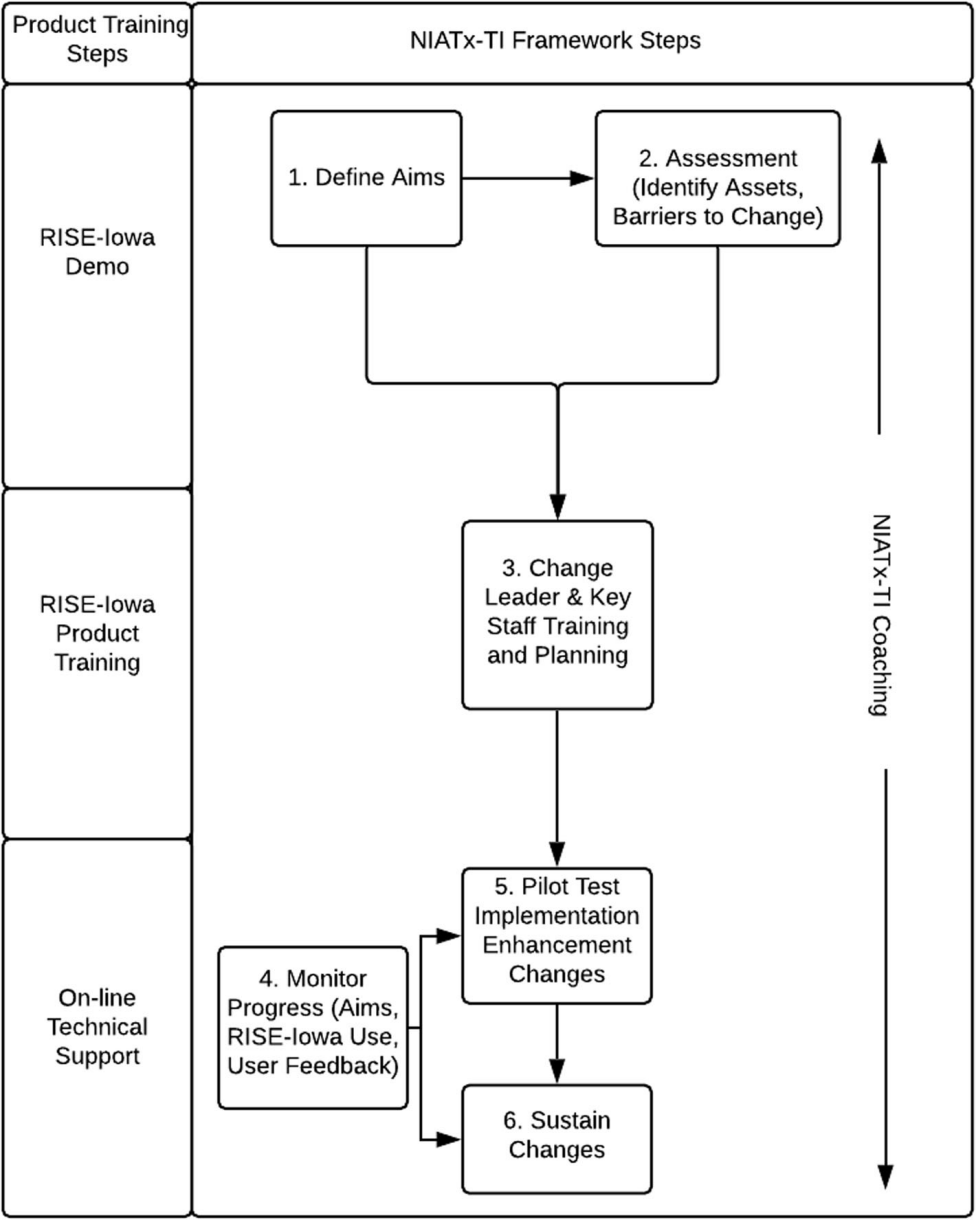
Product training and NIATx-TI framework overview

In addition to the above product training and on-line support, the second study arm has a NIATx-TI framework and NIATx-TI structural components. These components include deliberate and emergent planning features suggested in the planning literature, which will allow us to develop an implementation plan that incorporates the organization’s barriers/facilitators to adopting the technology and uses a validated organizational change model. Figure 2 presents the NIATx-TI framework:

- *Deliberate (planning) phase*: *step 1. Define aims*: Executive briefing on project setup and discussion of strategic and implementation aims for the organization. This occurs at the initial RISE-Iowa demo. *Step 2. Assessment* of assets and barriers via organizational change management survey (OCM). This is discussed in detail with the change leader for the intervention arm during the secondary RISE-Iowa demo.
- *Emergent phase*: *step 3. Change leader and change team training and planning* on how to modify organizational processes to address barriers to RISE-Iowa implementation (combined with secondary RISE-Iowa demo and RISE-Iowa product training visit). *Step 4. Monitor RISE-Iowa implementation* reviews progress on aims and gains real-time user feedback. The study team will provide weekly enrollment reports to the site coordinator on the number of patients enrolled in RISE-Iowa. *Step 5. Change cycles* (*or pilot tests*) to overcome barriers to implementing RISE-Iowa. *Step 6. Sustain changes*: Implement a plan to institutionalize gains. Additionally, approximately a year after RISE-Iowa is implemented, the coaches will conduct the sustainability survey verbally with the change team. The survey will help identify the sustainability of the implementation and lead to more productive change team meetings.

The *NIATx-TI structural components* involve:

- *NIATx coach*: an expert versed in technology adoption and organizational change who helps treatment organizations make, sustain, and spread RISE-Iowa adoption efforts. A NIATx-TI coach assists the organizations with applying the NIATx-TI framework, provides the training for this intervention, and provides feedback on the assessments during the session (Fig. 2, step 4) for arm 2 only. They conduct monthly calls with the RISE-Iowa implementation change leader to discuss data monitoring, including the weekly RISE-Iowa enrollment report and monthly RISE-Iowa usage report, and help problem-solve implementation issues.
- *Change team*: includes change leader, clinical leadership, select staff, and oversees RISE-Iowa implementation (Fig. 2, steps 5–7). The executive leader typically assigns the change leader. Together, they determine and recruit the change team members to include job functions that interact with the RISE-Iowa app. Change teams ideally meet weekly. The change team’s job is to be constantly vigilant for barriers to implementing RISE-Iowa, develop Plan-Do-Study-Act (PDSA) pilot tests to address identified barriers, and then implement any resulting changes to the RISE-Iowa implementation protocol.

The OCM and Klein’s Financial Resource Availability Inventory are administered to both arms through on-line surveys. However, the aggregated results of the OCM will only be provided to the NIATx-TI Arm during protocol development (Fig. 2, step 3) and progress monitoring (Fig. 2, step 5). Additionally, the sustainability survey will be conducted only with the NIATx-TI arm during sustain changes (Fig. 2, step 7).

### Timeline of study

During project months 1 to 16, the study team sought ethics approval, created materials for the intervention, recruited and collected baseline data on the organizations, and hired NIATx-TI coaches. In months 13 to 45, the study team implements the interventions and observes the organizations over an 18-month test period. In months 45–57, the organizations will be observed during the sustainability period. Months 55–60 will be used for data analysis and dissemination of research findings.

### Sample size

To determine sample size, we fit a linear mixed-effects model to the monthly results for the performance measures, percentage of eligible RISE-Iowa users, and frequency of RISE-Iowa use. These performance measures are calculated for each of the participating organizational sites and clustered by organization to estimate the NIATx-TI framework and product training effects. The power of the study design is determined by the anticipated standardized effect size based on effects experienced in a previous study conducted by one of our authors on e-health adoption in Iowa. This previous study found that NIATx-TI increased client technology use rates by 12.4% (increase of intervention success). This yields a Cohen’s *d* = 0.352 [67]. Additionally, intraclass correlation (ICC) among sites affects the power of cluster randomized trials [68]. An estimate of ICC is around 0.10 [22]. With a total of 5 or 7 organizations, with 23 organizational sites in each arm, where each site has an average 62 eligible patients, the study will achieve a power of .92 with a type I error rate of 0.05.

### Data collection and measures

Table 1 shows the frequency and data sources for all outcome measures. Our primary outcome is the (a) percentage of eligible patients who create an account on the RISE-Iowa app and (b) frequency of use by app user (in days). For the percentage who create RISE-Iowa accounts, data will be collected during the 18-month test period and 12-month sustainability period for that organization. Frequency of use data will be collected during the 18 months following the initial use of RISE-Iowa. The denominator for primary measure (a) will be based on the number of adult (age > 18) SUD patients. This data will be supplied by the Iowa Department of Public Health (IDPH) central data repository. The numerator for primary measure (a) will be the number of unique consumers who create accounts on the RISE-Iowa app. These, along with the frequency of RISE-Iowa use for primary measure (b), are collected in time-stamped log files and include when a patient accesses RISE-Iowa and the service(s) selected.

**Table 1:** Measures, sources, and tool frequency

Measurement	Measure source	Data source/frequency
**Descriptive statistics:**		
*Organizational traits*: admissions, rural v. urban	N-SSATS [72]	IDPH—Months 7, 20, 32
*Patient/counselor traits*: age, gender, ethnicity, and level of education (counselor only)	TEDS [73]	IDPH—Months 3-7, 20, 32
**REACH**: (a) % who create a RISE-Iowa account and (b) frequency of use in days	Gustafson [22]	RISE-Iowa server and IDPH—monthly, Months 8-44
**Mediation:**		
finance resource availability;	Klein [66];	Organizational survey—Months 7, 20, 32;
organization change management (OCM)	Gustafson [46]	Organizational and staff survey—Months 7, 20, 32
**Adoption:** % counselor using RISE-Iowa	Knight et al. [71]	Organizational survey and RISE-Iowa server—Months 7, 20, 32
**Effectiveness:** retention (length of stay)	Simpson et al. [69]	IDPH—monthly, Months 8-44

Within the two study conditions, we have the additional secondary measures of effectiveness, adoption, and mediation. Effectiveness: Retention rates will be measured using the Simpson measure of admission date to last therapeutic visit [69], because this length-of-stay measure has been positively associated with outpatient outcomes [70]. Adoption: Percent of counselors will be measured from the RISE-Iowa log-in data and compared against counselor numbers stated in the organizational survey, because counselor adoption of a technologies has been found to cause variation in an organization’s ability to apply a technology [32, 33, 66, 71]. Mediation: Organizational readiness (for technology use) will be monitored through the OCM tool [46]. Financial resource availability will be assessed through the completion of Klein’s Financial Resource Availability Inventory (*α* = .93) [66]. This inventory assesses the organization’s purchasing, training, and implementation resources. Additional questions will be added regarding reimbursement.

### Data analysis

A preliminary analysis of organizational data will compare baseline characteristics (admissions, rural v. urban, and gender/age/ethnicity) between organizations in each of the two intervention arms. The analysis will use the National Survey of Substance Abuse Treatment Services (N-SSATS) [72] and Treatment Episode Data Set (TEDS) [73] data definitions. This data will be collected from the Iowa Department of Public Health (IDPH) central data repository. Fisher’s exact and *t* tests (or Mann-Whitney tests) will be used to test for statistically significant baseline differences.

Initial exploratory analyses of RISE-Iowa use will assess standard summary statistics and graphical presentations at each level and across levels [74]. The data design represents a multi-site cluster randomized trial, and mixed-effects models (random effects due to organization; fixed effects due to study arm and time) will be used for data analysis. Mixed-effects models are also known as multilevel models. Primary outcomes (a) and (b) are nested within cluster (organizational site), and clusters are nested within organization. In a similar multilevel modeling framework, separate models will be applied for primary outcomes (a) and (b). Significant organizational, counselor, and patient differences between study conditions will be included in the models, along with factors included in the matched pairs sampling, as covariates to estimate the effect of NIATx-TI properly and efficiently. We will examine the NIATx-TI effect at each time point using cross-sectional multilevel models. We will also implement growth curve models across time that include the NIATx-TI effect, time, and NIATx-TI*time, as well as time-varying covariates. The best-fitting covariance structure will be determined before analysis based on the Akaike Information Criterion (AIC) and Bayesian Information Criterion (BIC) [75].

We will examine the mediating effects of organizational factors of percentage of counselors using RISE-Iowa, organizational readiness (via the OCM), and financial resource availability (via Klein’s inventory), through a causal mediational analysis, using data from mid-test, end of test, and end of sustainability (post-test) periods. These factors will be analyzed using mixed-effects models. Through a mediation analysis [76, 77], we can estimate the direct (RISE-Iowa use with NIATx-TI and product training) and indirect effects (adoption and implementation factors) of each implementation arm on RISE-Iowa use. We will test each potential mediator’s mediation effect at each time point and across time. The R package “mediation” will estimate the causal mediation effects, examine moderated mediation effects, and conduct sensitivity analysis [78].

The qualitative portion of the study will support the specific aims by exploring how NIATx-TI and product training are implemented, how implementation affects RISE-Iowa use, and how a staff user reacts to NIATx-TI, product training, and RISE-Iowa. During the study, interviews from each of the organizations (*n* = 12) will occur at months 40 to 45 (at the end of the test phase) and months 53 to 57 (at the end of the sustainability phase). A randomly selected site from each organization will be used for the interviews in each phase to gain a retrospective understanding of what occurred during implementation. The interviews will include closed- and open-ended questions designed to explore participants’ experiences within each arm and the perceived effectiveness of each approach in assisting with implementing RISE-Iowa. The closed-ended questions will be based on elements of the Consolidated Framework for Implementation Research, developed by Damschroder et al. [79], that will address (1) intervention characteristics, (2) outer setting, (3) inner setting, and (4) process. The additional open-ended questions will allow for the discovery of other factors affecting NIATx-TI, product training, and RISE-Iowa implementation. The information collected will inform our understanding of implementation assets, barriers, and potential revisions for each approach. Interviews of up to 30 min will be conducted with the organization’s executive director, the site’s clinical director, the RISE-Iowa site coordinator, and a randomly selected full-time counselor that has been at the organization for at least 6 months. Four interviews per organization will result in *n* = 48 interviews per phase. The qualitative analysis will produce a description of the technology implementation process. It will encompass grounded explications of the theoretical concepts that influenced the development of the NIATx-TI framework. This description will enhance our understanding of how the NIATx-TI framework works, promoting valuable insights that can be applied to future dissemination of mobile e-health applications.

Any research protocol today needs to consider the impact that COVID-19 may have on patients, staff, and SUD organizations. Therefore, the interviews will also ask how staff and leaders are responding to the virus and what perceived effect the virus is having on RISE-Iowa adoption and use. Many treatment agencies have had to replace face-to-face treatment with virtual counseling. While RISE-Iowa does not include a virtual counseling service, some of the enrolled treatment agencies have started to use RISE-Iowa to backfill weekly virtual counseling services. For example, virtual sessions can include homework assignments that would require the use of RISE-Iowa between sessions.

### Ethics

The study received external approval from Independent Review Board Services (IntegReview IRB).

### Trial status

Currently, the trial’s recruitment phase is complete, much of the baseline data has been collected, and the implementation period has started.

## Discussion

More than 20 million adults aged 18 or older struggle with a SUD each year in the USA. Yet, only around a tenth seek SUD treatment [4, 5, 80, 81], and even fewer achieve long-term sobriety. E-health applications in healthcare have proven to be effective at sustaining treatment and reaching patients’ treatment otherwise would have missed. However, e-health adoption rates in healthcare have been historically poor. In SUD care, using a treatment and recovery mobile app in conjunction with outpatient SUD treatment is highly innovative and has become an increasingly popular option for SUD treatment providers amid the COVID-19 pandemic. Additionally, thousands of studies and hundreds of theories exist on implementing innovations [28, 82–84], and the number of evaluations of e-health applications is expanding rapidly. Yet, beyond those mentioned above, we could not find any RCTs that empirically examined the relative effectiveness of competing implementation strategies. This study builds on the work described above and will, to our knowledge, be the first to compare an evidence-based implementation strategy (NIATx-TI) with a widely used implementation strategy (product training) in a randomized trial. Therefore, we implement an e-health treatment framework into SUD outpatient therapy to assess the impact of NIATx-TI in overcoming barriers to mobile e-health technology and patient outcomes.

## Data Availability

Not applicable

## Abbreviations

A-CHESS: Addiction Comprehensive Health Enhancement Support System
AIC: Akaike Information Criterion
BIC: Bayesian Information Criterion
CHESS: Comprehensive Health Enhancement Support System
ICC: Intraclass correlation
IDPH: Iowa Department of Public Health
IntegReview IRB: Independent Review Board Services
IRHIT: Iowa Rural Health Information Technology Initiative
NIATx-TI: Network for the Improvement of Addiction Treatment–Technology Implementation
N-SSATS: National Survey of Substance Abuse Treatment Services
OCM: Organizational Change Management
PDSA: Plan Do Study Act
RISE-Iowa: Recovering Iowans Supporting Each other
SDT: Self-Determination Theory
SUD: Substance Use Disorder
TEDS: Treatment Episode Data Set
